# Absence of the Right Common Iliac Vein with the Right Internal Iliac Vein Arising from the Left Common Iliac Vein: Case Report

**DOI:** 10.7759/cureus.4575

**Published:** 2019-04-30

**Authors:** Karishma Mehta, Joe Iwanaga, R. Shane Tubbs

**Affiliations:** 1 Clinical Anatomy, Seattle Science Foundation, Seattle, USA; 2 Medical Education and Simulation, Seattle Science Foundation, Seattle, USA; 3 Neurosurgery, Seattle Science Foundation, Seattle, USA

**Keywords:** anatomy, common iliac vein, cadaver, variation

## Abstract

The common iliac vein arises from the internal and external iliac veins and drains into the inferior vena cava, bilaterally. Historically, many anatomical variants of the common iliac vein have been studied. To our knowledge, we discuss a unique specimen, which presents with an absent right common iliac vein and a right internal iliac vein draining into the contralateral common iliac vein. It is important that we understand the anatomic variations of the pelvic venous system to prevent iatrogenic injury to patients or misdiagnosis.

## Introduction

The common iliac vein is formed by the internal and external iliac veins, which join at the level of the fifth lumbar vertebra, anterior to the sacroiliac joint. The left common iliac vein is longer than the right, as both common iliac veins unite on the right side to form the inferior vena cava near the L5 lumbar vertebra. Each common iliac vein receives drainage from the iliolumbar and lateral sacral veins while the left side also receives drainage from the median sacral vein [1]. The majority of the pelvis drains into the systemic venous system via the internal iliac vein while the femoral vein drains and is confluent with the external iliac vein at the inguinal ligament [2].

Gray notes a possible variation in which the left common iliac vein ascends toward the kidney, rather than traveling to the right [1]. It then joins with the left renal vein, crosses over the aorta, and drains directly into the inferior vena cava. In this scenario, the right and left common iliac veins are joined together by a small communicating branch. Morris states that there are four chief variations in the common iliac veins: 1) the common iliac vein can be doubled, 2) one may be absent, in which case the internal and external iliac branches can drain into the contralateral common iliac vein, 3) the right and left internal iliac veins can form a common trunk that drains into the external iliac veins and inferior vena cava, and 4) the middle sacral vein can split and drain into both right and left common iliac veins [3-4].

Herein, we report a case in which the right common iliac vein was absent and the right internal iliac vein drained to the contralateral side.

## Case presentation

**Figure 1:**
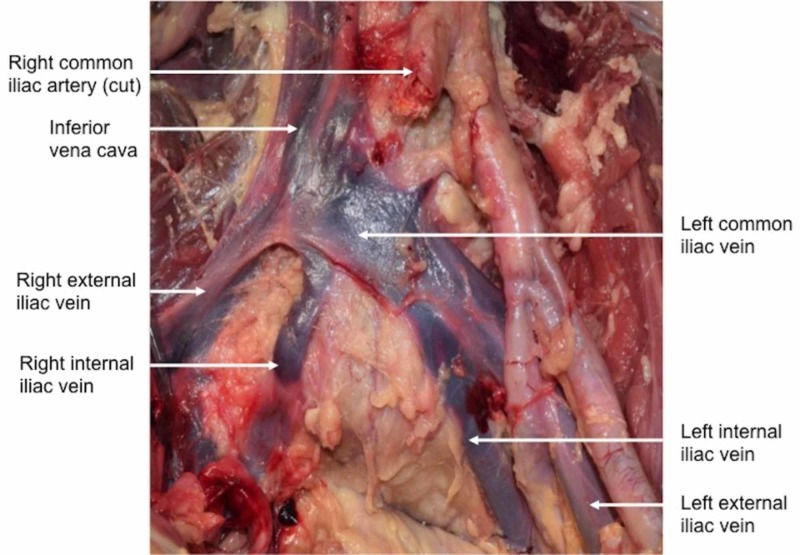
Anterior view of the case reported herein. Note the right internal iliac vein draining into the left common iliac vein.

During routine dissection of the posterior abdominal wall, the right common iliac vein was absent (Figure 1). The specimen was from an elderly male who was 92-years-old at death. On the right side, the bifurcation of the inferior vena cava was formed by the right external iliac vein and the left common iliac vein. The right internal iliac vein was found to drain into the proximal left common iliac vein near the midline (Figure 1). This vessel traveled into the lesser pelvis by coursing over the sacral promontory and then had a normal distribution in the pelvis. It coursed deep to the internal iliac artery and was 12 mm in diameter. For comparison, the left internal iliac vein was 14.8 mm in diameter. From origin to its first branches in the pelvis, the right internal iliac vein was 9 cm in length and the left internal iliac vein was 6.8 cm long. No other anatomical variations were noted in the abdominopelvic region. The overlying distal abdominal aorta and iliac vessels were found to have atherosclerosis, but there were no other pathological findings noted in the abdominopelvic cavity.

## Discussion

Looking at the embryological development of the common iliac vein and its subsidiaries may provide insight into the potential causality of these types of variations. During embryogenesis, the cardinal veins provide most of the venous drainage into the sinus venosus, with the anterior cardinal veins draining the cranial portion of the embryo and the posterior cardinal veins draining the caudal portion. Most of the posterior cardinal veins regress except the iliac anastomosis portion and the subcardinal and supracardinal portions. The supracardinal portion will eventually give rise to the inferior vena cava while the iliac anastomosis will become the common, external, and internal iliac veins. Any malformation in this process can result in the agenesis of the common iliac vein.

Previous studies have shown variations of the common iliac vein and its subsidiaries. Both Yahyayev et al. and Sierre and Garriga described an absent common iliac vein with the femoral vein draining into the contralateral side [5-6]. Yahyayev et al. report a case of agenesis of the right common and external iliac veins in a seven-year-old boy. Subsequently, the right femoral vein drained into the left femoral while the right internal iliac vein drained directly into the inferior vena cava [5]. Sierre and Garriga reported a three-year-old girl with an absent left common iliac vein, in which the left femoral vein emptied into the right femoral vein. Both patients were asymptomatic besides having suprapubic varicosity [6].

Lotz and Seeger and Morita et al. conducted imaging studies in which alternative anatomies of the internal iliac vein were studied [7-8]. Lotz and Seeger mentioned five possible variations of the internal iliac vein. Of note, two out of 100 patients demonstrated a connection between the right internal iliac and left common iliac veins, with no mention of an absent right common iliac vein using epidural venograms [7]. Morita et al. reported six variations via contrast-enhanced CT. Eleven percent of their patients exhibited a type D formation, in which a separate and duplicate trunk of the right internal iliac vein fed into the left common iliac vein [8]. Biswas and Singh reported a case in which a 47-year-old female had agenesis of the common iliac vein on either side. Instead of the normal configuration of veins, she had the right and left external and internal iliac veins all draining directly into the inferior vena cava [9]. Lastly, Huban et al. discussed a case in which a cadaver, 55-years-old at death, exhibited a communication between the right internal and left common iliac veins. However, the right common iliac vein was present [10]. Iwanaga et al. reported a case of absence of the right common iliac vein in a cadaver with an L-shaped kidney and another case of absence of the right common iliac vein in a cadaver with bilateral malrotated kidneys. The right internal iliac vein directly drained into the left common iliac vein in both reports [11-12]. Maldevelopment of the abdominopelvic cavity during fetal life might affect the formation of the iliac vessels.

Anatomical variants are important to recognize, as they can aid in the identification of possible disease states and in the prevention of exacerbation. For example, “supine hypotensive syndrome” occurs due to inferior vena cava (IVC) obstruction leading to an inadequate venous return to the heart. This syndrome especially manifests in pregnant women during the third trimester, as their cardiac demand is at its highest. In cases of IVC obstruction, the body uses collateral venous systems in order to return blood back to the heart. The superficial collateral system, consisting of the anastomosis of the external iliac vein and inferior epigastric vein, connects the drainage of the abdomen to the superior vena cava (SVC), bypassing the IVC altogether [13]. If however, an anatomical variant is present in a patient with this pre-existing condition, this collateral system is compromised and further intervention could be required. 

Recognizing anatomical variants can also ensure treatment efficacy. Patients with chronic venous insufficiency (CVI) experience pain with claudication due to increased pressure in postcapillary vessels. Understanding the exact topography, including the anatomical variants of the venous system, can aid in the placement of the venous valve implant in CVI therapy [14].

Although a routine dissection technique was used in our research, an alternative method has been documented using air dissection. Iwanaga et al. also described a novel technique in vein dissection in which air was insufflated first in the IVC and then its tributaries. They noted a case in which the right internal iliac vein was only visualized using air dissection rather than classic methods [15]. Use of air dissection enhances the detection of anatomical variants and could potentially lead to more-efficient diagnosis and disease prevention.

## Conclusions

We present a variation in which the right common iliac vein is absent along with the right internal iliac vein feeding into the contralateral common iliac vein. It is important that we document such venous anomalies in order to prevent the exacerbation of disease states and aid in interventional procedures.
